# Whose Responsibility Is It? A Community-Level Situational Analysis of Oral Health Care in Amsterdam

**DOI:** 10.1177/23800844251332227

**Published:** 2025-05-02

**Authors:** S. Begovic, M.W. van der Linden, K. Rosing, L.E. de Almeida, M. Lorenz, S. Listl, M.H. van der Veen

**Affiliations:** 1 Department of Oral Public Health, Academic Centre for Dentistry Amsterdam, University of Amsterdam and VU University Amsterdam, The Netherlands; 2 Section for Oral Health, Society and Technology, Department of Odontology, University of Copenhagen, Denmark; 3 Section for Oral Health, Heidelberg Institute of Global Health, Heidelberg University Hospital, Heidelberg, Germany; 4 Department of Dentistry–Quality and Safety of Oral Healthcare, Radboud University Medical Center–Radboud Institute for Health Sciences (RIHS), Nijmegen, the Netherlands; 5 Department of Preventive Dentistry, Academic Centre for Dentistry Amsterdam, University of Amsterdam and VU University Amsterdam, The Netherlands; 6 Department of Paediatric Dentistry, Academic Centre for Dentistry Amsterdam, University of Amsterdam and VU University Amsterdam, The Netherlands

**Keywords:** oral health, quality improvement, oral health policy, health accessibility, qualitative research

## Abstract

**Background:**

DELIVER (DELiberative ImproVEment of oRal care quality) is a multinational project funded under the EU’s Horizon Europe program that aims to improve the quality of oral health care at the practice, community, national, and international levels. It is important to understand the current situation of oral health care quality to make improvements possible. This study aimed to map oral health care priorities among stakeholders in Amsterdam, the Netherlands, and to describe how these stakeholders interacted to improve the quality of oral health care at the community level.

**Methods:**

A situational analysis approach was used to collate data from desk research and semi-structured interviews with key informants. Interview transcripts were analyzed and grouped into main themes and subthemes using inductive coding. A situational map, a social worlds/arenas map, and a positional map were constructed to represent the community-level situation of quality improvement of oral health care.

**Results:**

Interviews were conducted with 10 professional stakeholders (5 social/welfare workers, 3 health care professionals, 1 public health professional, and 1 municipality policy maker). Stakeholders described prioritizing at least basic oral health care and stated that it should be accessible for everyone. Other priorities included a need for simplified access to oral health care and strengthened social support. While stakeholders agreed that people should not rely on emergency funds and volunteers, they felt that it was unclear which organizations or individuals were responsible for determining access to oral health care. This led social/welfare organizations to feel a sense of responsibility and offer informal care solutions.

**Conclusion:**

There was consensus among stakeholders about the need for social support and simplified access to oral health care for citizens. Stakeholders also emphasized the lack of clarity about who was responsible for oral health care and quality improvement at the community level, which highlighted the urgent need for improved governance, allocating responsibilities for oral health care quality improvement to all parties operating at the community level.

**Knowledge Transfer Statement:**

This study mapped the current practice of oral health care quality in Amsterdam, the Netherlands, through a situational analysis as a crucial starting point for enhancing quality improvement of oral health care at the community level. It underscored the need for clarity about responsibilities and provided insights for oral health care providers, social and welfare workers, policy makers, and researchers that could support research and policy formulation targeted at underserved populations, involving multiple stakeholders.

## Introduction

Oral diseases affect nearly half of the European population (Patel 2012). Despite their high prevalence, statutory coverage of oral health care (OHC) across European countries is limited by restricted service packages, resulting in high private funding compared with other health services (European Observatory on Health Systems and Policies 2022). According to a recent working definition, the quality of OHC comprises 7 domains: patient safety, effectiveness, efficiency, patient-centeredness, equitability, timeliness, and access to care (Righolt et al. 2020). According to Righolt et al., a working definition allows for adaptability and encourages further exploration and collaboration in the pursuit of quality improvement (QI). This recently proposed working definition of quality of OHC suggests that OHC should be accessible and available to everyone seeking care, independent of personal characteristics such as socioeconomic status and the patient’s health status (Righolt et al. 2020). In the Netherlands, it is mandatory for all residents to have basic health insurance (Rijksoverheid 2024). Such insurance covers essential medical care, such as visits to general practitioners and most types of specialist care (Rijksoverheid 2024). OHC is generally not included in coverage from the basic health insurance (Zorginstituut Nederland 2024). However, exceptions exist, such as essential OHC provided by an oral and maxillofacial surgeon and regular dental checkups for children until the age of 18 y. For adults, it is possible to take additional dental insurance on top of basic health insurance (Zorginstituut Nederland 2024), which can vary in coverage and costs between health insurers. The role for OHC within public health care is limited. The Public Health Act (PHA; Dutch: Wet Publieke Gezondheid) in the Netherlands is a legal framework that governs public health policy, such as control of infectious disease crises (Overheid Wettenbank 2023). It involves health-protecting and health-promoting measures for the general population or specific groups in society, including the prevention and early detection of diseases (Overheid Wettenbank 2023; RIVM 2024). While it does not directly address OHC, it supports public health initiatives that can affect oral health, such as health education campaigns. In the Netherlands, social inequalities in access to OHC among older adults have been shown to be higher than in several other European countries (Shen and Listl 2018). According to the Organisation for Economic Co-operation and Development, 3% of the Dutch population (435.122 people) avoid OHC for financial reasons (OECD 2019). However, within the lowest income quintile, in the Netherlands, this figure rises to 12% (348,098 people; OECD 2019), illustrating a disparity within the country by income. This inequality and high private funding are detrimental to the quality of OHC.

Understanding a problem is considered the first relevant step in decision-making processes to optimize oral health (Listl et al. 2023). Individuals in the Netherlands who lack access to OHC for financial reasons or because of the absence of supplementary dental insurance may be solely dependent on informal care solutions. Informal care solutions focus on basic OHC services such as pain reduction and reinstating oral function, facilitated by social and welfare organizations who feel responsible for accessible health care for everyone (Begovic et al. 2023). Informal care initiatives to improve the accessibility of OHC frequently originate at the community level. Examples are emergency funds supported by municipalities, collective health insurances for minimum-income groups, and informal networks dedicated to OHC (Dokters van de Wereld 2024; Fonds Bijzondere Noden Amsterdam 2024; Gemeente Amsterdam 2024).

Investigating possibilities for OHC improvement is at the center of the Deliberative ImproVEment of oRal care quality (DELIVER) project (Listl et al. 2024). In DELIVER, with partners from 7 European countries, a mixed-methods research approach is used to convert deliberative dialogues into meaningful support for improving the quality of OHC. This involves a situational analysis of the status quo of QI across Europe with a focus on several levels, including the practice, community, national, and international levels. The purpose of this study was to map (oral) health care priorities at the community level in Amsterdam and to describe how context-relevant stakeholders interact to improve the quality of OHC at the community level. Understanding the interconnections among actors within the broader social and welfare domain can help improve the quality of OHC especially for vulnerable, underserved, and marginalized populations.

## Methodology

### Study Design

This study was a community-based exploratory qualitative study. It was part of the first phase (a situational analysis of the status quo of QI) within the HORIZON Europe DELIVER project (Listl et al. 2023). The situational analysis method (Clarke et al. 2017) was used to investigate and map the situation of QI of OHC at the community level in Amsterdam, the Netherlands. A semi-structured interview guide was developed by authors in collaboration with DELIVER project members, based on the Consolidated Framework for Implementation Research (Damschroder et al. 2022). The interview guide was created based on the authors’ collective expertise and previous knowledge in the field. The methodology and results of this study were described by following the Standards for Reporting Qualitative Research (O’Brien 2014).

### Ethical Considerations

The research protocol for this study was approved by the Ethics Committee of the Medical Faculty of the University of Heidelberg (S-089/2023). The research protocol for this community-based exploratory qualitative study was deemed not subject to the Medical Research Involving Human Subjects Act by the Academic Centre for Dentistry Amsterdam Institutional Review Board (ACTA-ETC protocol No. 2023-26070).

### Data Collection and Analysis

Following the situational analysis method (Clarke et al. 2017), the following steps were taken iteratively: (1) desk research, (2) recruitment of key informant interview respondents and data collection, (3) data analysis, and (4) mapping (creating a situational map, social world/arenas map, and positional map).

### Desk Research

**Figure 1.**
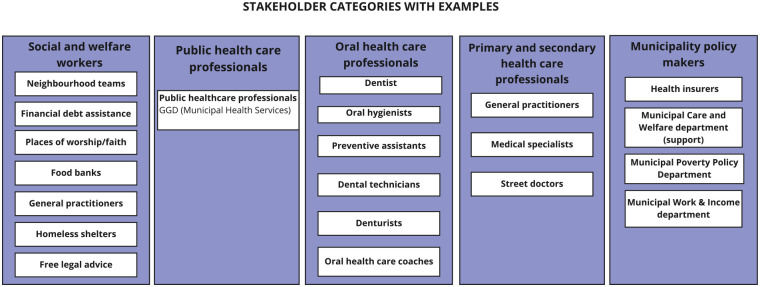
Ordered situational map of actors involved in quality improvement efforts within oral health care at the community level in Amsterdam, the Netherlands.

To identify stakeholders who could have possibly been involved at the community level, information was used from an earlier report from our group as commissioned by the Ministry of Health, Welfare and Sports (Begovic et al. 2023). Briefly, the report described an overview of social organizations in the context of unwanted avoidance and barriers to accessibility of OHC (Begovic et al. 2023). Further insights were gained through “gray literature” (news and opinion articles in newspapers and tv documentaries; Dokters van de Wereld 2024; Fonds Bijzondere Noden Amsterdam 2024; Gemeente Amsterdam 2024). A preliminary list of candidate organizations and individuals was compiled to include the following categories: social and welfare workers, (oral) health care professionals, public health professionals, and municipality policy makers. The relevance of candidate organizations and possible stakeholders was derived from the existing literature (Everaars et al. 2018; Mitchell et al. 2023). Stakeholder categories were summarized based on the situational analysis methodology (Clarke et al. 2017) in an adapted ordered situational map of stakeholders involved at the community level in Amsterdam, the Netherlands (Fig. 1).

### Recruitment of Interview Respondents and Data Processing

Stakeholders who matched one of the categories in Figure 1 and who were involved in community organizations around Amsterdam were considered eligible for semi-structured interviews. The exclusion criteria were stakeholders without any direct involvement or experience relevant to oral health or community-based initiatives in the Amsterdam area. Respondents were recruited with a mix of purposive (Palinkas et al. 2016) and convenience sampling methods (Andrade 2021) until at least 1 respondent from each stakeholder category was interviewed. Interviews were conducted by S.B. and I.N. following the interview guide (Appendix: Interview Guide) and were held online or in person. The interviews were conducted in Dutch to ensure that participants could express themselves in their native language. We then used DeepL translator (DeepL Translator 2017) as a language translation tool to translate transcripts into English, followed by manual reviews of the translation. Interviews were recorded to enable verbatim transcription if respondents provided written informed consent for audio recording. Otherwise, field notes were taken by the interviewer. Recruitment of interview respondents was discontinued when no new themes could be identified.

### Data Analysis

After verbatim transcriptions and field notes were completed, data were analyzed using qualitative analysis software Atlas.ti (version 28). Grounded theory (Khan 2014) was used as a method for thematic analysis (Braun and Clarke 2006). In line with grounded theory methodology (Strauss and Corbin 1998; Khan 2014), an inductive approach was used for initial coding, followed by axial coding, selective coding, and the identification of main themes and subthemes. Axial coding systematically connected categories and subcategories to reveal how these elements interrelate, while selective coding refined these linkages into cohesive themes. This multistep process was well suited to our research question as it ensured that emerging categories and themes were grounded in the data and reflective of the complexities inherent in the phenomena being studied. Analysis was performed by author S.B., and coding was discussed and verified with author M.v.d.V. The situational analysis methodology, developed by Adele Clarke (Clarke et al. 2017) was used, enabling us to assess the complex situation through the creation of a situational map, a social worlds/arenas map, and a positional map.

### Researcher Characteristics and Reflexivity

Before analyzing the data, S.B. reflected on interviews with a reflection form (Appendix: Interview Reflection Form). Reflection allowed to improve interview skills and limit subconsciously influencing interview questions (McGrath et al. 2019). S.B., who has a background in dentistry, had no professional or personal relationship with the interview respondents. To improve the credibility of the data analysis, coding was discussed and verified between S.B. and M.v.d.V. Themes were discussed and feedback was sought from all authors.

## Results

Ten semi-structured interviews were conducted between May and July 2023 with at least 1 respondent from each stakeholder category (Table 1). Five interviews took place online (MS Teams and Zoom), 2 at the stakeholder’s location, and 3 at the Academic Centre for Dentistry in Amsterdam. From the 10 interviewees, 3 worked at the policy or organizational level for their organization and 7 had direct contact with clients on a daily basis. The interview duration ranged between 34 and 74 min, with an average of 54 min. Audio recordings were made for 9 of 10 interviews; for 1 interview, written notes were used for analysis because no audio recording was available.

**Table 1.** Respondents by Stakeholder Category and Organization.

Respondent Number	Stakeholder Category	Organization
R1	Public health care professionals	Municipal Health Services Amsterdam (GGD)
R2	Municipality policy makers	Municipality of Amsterdam, Collective Health Insurance for Minimum Income Groups and Poverty Reduction (Gemeente Amsterdam, Collectieve Zorgverzekering voor Minima en Armoedebestrijding)
R3	Oral health care professionals	Anxiety dentist from SBT Special Dentistry (Stichting voor Bijzondere Tandheelkunde)
R4	Oral health care professionals	Dentist with affinity for special dentistry
R5	Primary and secondary health care professionals	Social nurse at Streetdoctors
R6	Social and welfare workers	Homeless shelter (de Regenbooggroep)
R7	Social and welfare workers	Emergency Fund (Fonds Bijzondere Noden Amsterdam)
R8	Social and welfare workers	Buurtteam branch (social assistance for care, housing, health, income, financial issues, meeting people, and safety)
R9	Social and welfare workers	Médicins du Monde (Dokters van de Wereld)
R10	Social and welfare workers	DOCK Welfare institution

### Situational Map

**Figure 2.**
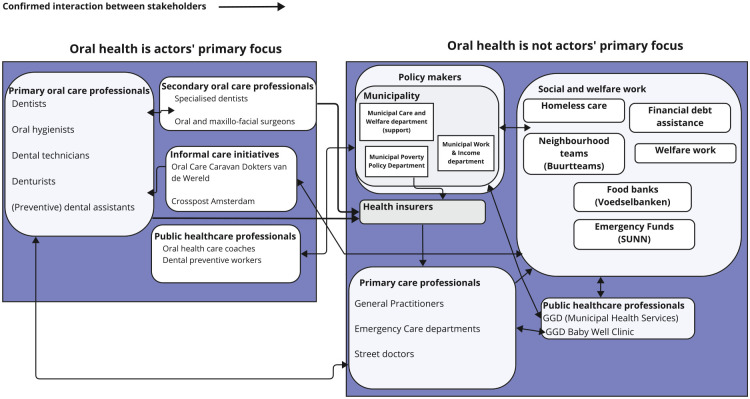
Situational map: relations between actors involved in the quality improvement of oral health care at the community level in the Netherlands.

A situational map (Fig. 2) was created to visualize relations and priorities among actors involved in OHC at the community level, based on data from interviews with stakeholders. On the left side of the situational map, actors for whom oral health is a primary focus were grouped together. This group included OHC professionals, public health care professionals, and informal care initiatives focused on providing OHC treatments. On the right side, actors for whom oral health was not the primary focus were listed. This group included primary care professionals and public health care professionals who were not preventive dental workers or OHC coaches, policy makers, and social/welfare workers. However, their primary objectives intersected with OHC. For example, social/welfare workers had clients with urgent OHC needs who asked for help in accessing OHC. This motivated social/welfare workers to give attention to oral health and to collaborate with other stakeholders. As shown in the relational situational map (Fig. 2), most interactions occurred within the group of stakeholders whose primary focus was outside the oral health area (Fig. 2, right panel), for example, interactions between public health care professionals and social and welfare workers and interactions between municipality workers and social workers. Fewer interactions, albeit important, occurred between (oral) health care professionals (Fig. 2, left panel) and policy makers, health insurers, and social/welfare workers (Fig. 2, right panel). For example, emergency funds for the treatment of acute dental problems were often requested through social and welfare workers, who needed a treatment plan from primary oral care professional to be able to request an emergency fund for their clients.

### Themes

The discussed topics of the interviews were analyzed and categorized in 8 themes.

#### Theme 1: Policies

The Municipal Health Services (MHS), known as *Gemeentelijke Gezondheidsdienst* in the Netherlands, reported to be aware that oral health is within their responsibilities as part of implementation of the PHA. Despite the clear description of the PHA (Overheid Wettenbank 2023; RIVM 2024), professional stakeholders in Amsterdam did not describe policies for oral health promotion. The MHS reported that they try to address oral health within other frameworks, such as healthy weight, nutrition, and poverty reduction:

When I come across oral health within that framework which is about health and lies within the possibilities of municipal influence, I do try to raise and put on the agenda. (R2, municipality policy maker)

When external policies were discussed, “the system” was described as unfair and not supportive for access to OHC. Stakeholders expressed unclarity about who has ownership and responsibility of OHC accessibility. According to them, not all people had equal rights, and municipal regulations for emergency OHC varied widely. Stakeholders also stated that access to OHC should not rely on the individual but on a supportive structure that works:

The question is with whom the responsibility lies. With the social domain? Or with the insurers, because it is about treatment? Those are system things. In any case, it does not lie with the individual, there has to be a structure that works. (R1, public health care professional)

#### Theme 2: Cooperation in communities

Most stakeholders mentioned collaborating with each other and having direct contact with citizens in their work. Social workers reported being easily accessible within neighborhoods and maintaining close contact with citizens. In contrast, the municipal department for poverty reduction indicated having no direct contact with citizens. However, the municipality engages citizens in policy discussions through a participation council representing individuals receiving welfare benefits. OHC providers primarily collaborated within their stakeholder group or with health insurers but had fewer interactions with the social sector. According to non-OHC providers, working with OHC providers can raise awareness of oral health within their organizations:

I’m pleasantly surprised with ACTA (dental faculty) suddenly being here more. Since then, I’ve maybe also been paying more attention to the mouth than before, so I really like that.’ (R6, social and welfare worker)

Working with OHC providers could also lead to frustrations. For example, a dentist refused to treat patients after 1 no-show appointment, after a social worker had sent these clients. More understanding from OHC providers was wished for by social workers:

This population generally struggles with scheduled appointments and finds them stressful. It’s best not to place too many expectations on them in this regard, as they frequently have difficulty adhering to rigid scheduling. For example, I contacted the dentist to explain the situation: this individual is homeless, making appointments is especially challenging for him. The answer was the gentleman is not welcome in the dental office anymore because he left us out in the cold [did not show up to the appointment]. (R5, primary health care professional)

When it came to emergency funds, a desire for greater involvement of OHC providers with patients’ social situation was expressed. The perspective from one social and welfare worker (R7) raised concerns about a potential lack of engagement from OHC providers once an invoice is sent to the emergency fund and payment is secured:

I wonder if, when a dentist proposes a treatment and anticipates it will be covered by our [emergency] fund, they just think, “it will be paid for later,” and not concern themselves further about the social situation of the patient. For the dentist, it’s merely an invoice—handled by administrative systems—so there is no additional follow-up from their side. (R7, social and welfare worker)

#### Theme 3: Learning climate and culture within organizations

Most stakeholders described their organizations as open work environments, approachable and facilitating brainstorming and evaluation opportunities. Stakeholders explained that they get inspired from other organizations and initiatives and that they know how to approach each other. However, stakeholders felt that there was not always enough time to implement new initiatives:

Well, my experience is from what I see around me, there is a lot of room for new initiatives and that sometimes that becomes a bit too much. As a debt relief worker, you just have your regular work. And of course, we try to adapt that more and more to the people who need it, but it’s quite obvious what we have to do. (R8, social and welfare worker)

Stakeholders also described negative experiences. For example, the organization for special care dentistry explained that they run behind in digitalization, which has their current priority and leaves little room for new initiatives. Other organizations mentioned that their organizations budget had reduced. The respondent from the MHS expressed uncertainty about the organization’s ability to support new initiatives:

The MHS aims to be flexible; management would certainly say so. But whether it truly is, can vary. If you want to do new things, you also have to let go of other activities that are not strictly necessary. For instance, focusing on more applied research and policy development, rather than long PhD projects. (R1, public health care professional)

Even though negative aspects existed, all stakeholders showed motivation to work for their organizations. Special care dentists described being attracted to their job because they treated patients who needed more than dental work, such as guidance in behavior and anxiety reduction, and this challenges them intellectually and beyond their average dental skills. Medical volunteers described that they offer free services to those in need and do so to give back to society:

I also think it’s about awareness. That it’s not just about making money for themselves, but that they really do want to mean something more in society. (R9, social and welfare worker)

#### Theme 4: Oral health prioritization

Of all stakeholders, OHC providers were the only stakeholders to describe OHC as the primary focus of their organizations. Other stakeholders provided examples in which they did give attention to oral health, but it became a topic only when relevant to other themes, such as specific client needs:

Oral healthcare is not routinely addressed. In the past, we followed guidelines with a comprehensive list of topics to discuss, but people weren’t very receptive to that. Now, our approach is more interactive and guided by individuals’ questions, reflecting current practices. For instance, if a mother breastfeeds very frequently on demand, we emphasize the possible implications for her child’s dental health. (R1, public healthcare professional)

Stakeholders acknowledged the importance of prioritizing oral health. The Municipality of Amsterdam emphasized it within poverty reduction initiatives, such as the collective health insurance for low-income populations. The Emergency Fund recently prioritized OHC due to increasing requests for financial support to access OHC. Social workers acknowledged oral health as a relevant issue within their work, noting that many clients struggled with poor oral health. A key factor in prioritization was whether stakeholders felt they had sufficient knowledge and resources to address and discuss the issue:

To work with oral health within this organization, I think I have enough knowledge, and I can participate sufficiently. My brother is a dentist, and I was a project leader healthy lifestyle, with an oral care project under that. Well, I just went through some things with him. At some point you become a kind of expert in coordinating oral care for vulnerable target groups. (R9, social and welfare worker)

Barriers to prioritizing oral health were also identified. Stakeholders mentioned that oral health was not important enough for their organization, making it difficult to allocate more attention to it. In addition, oral health issues were often overshadowed by more pressing client concerns. Social workers, dealing with a wide range of topics, explained that limited time and higher-priority issues often prevented discussions about oral health.

#### Theme 5: Patient needs and resources

Stakeholders were not optimistic about the accessibility of OHC. They pointed to citizen factors and health system factors as reasons for this difficulty. Citizen factors mentioned included knowledge on when to visit the dentist. Citizens often had complex problems to deal, with and stakeholders believed that in current society social problems have become even more complex. Financial barriers, insufficient health insurance coverage, and fear are common reasons to keep people away from the OHC provider. This often comes with a feeling of guilt and shame for their entire situation and oral health condition:

Well, there is shame of having ugly teeth, mouth odor. That can go a long way; that people have trouble with work anyway, or partners having trouble with that person. That is shame. There is also a lot of shame in going to the dentist. They are a bit afraid of your judgement. (R3, OHC professional)

Stakeholders believed systemic factors contributed to limited access to OHC and stated that being unable to receive OHC is a huge injustice. A citizens’ financial position and health insurance determined their ability to visit an OHC provider. However, beyond financial barriers, the complexity of treatment costs and the variety of health insurance options were seen as additional obstacles. Emergency funds, which had strict criteria and covered only basic care, explained that their request procedures were time-consuming and restrictive, excluding treatments such as periodontology, as it is not considered basic care. This led stakeholders to describe citizens who struggled to navigate the complex health system. According to professional stakeholders, citizens needed more information and knowledge about the health system, beneficial health behavior, insurances, and emergency resources:

What does have a big impact on whether someone can get oral care is the health system. For example, if something needs to be done at the surgeon’s, it is generally covered under the basic insurance, but dental costs are not covered; and who has supplementary dental insurance if you have little money? So that has a big impact. (R8, social and welfare worker)

Stakeholders agreed that at least basic OHC should be available and accessible for everyone. According to them, this care should be structural and not incidental:

It is about what is needed for the target group, when it comes to adjustments to the insurance. (R2, municipality policy maker)

An important factor related to foregoing OHC shared by stakeholders was that some people found to it difficult to access OHC without social support. Stakeholders were aware that social support came from professionals who guided patients to their appointment or someone from their personal social network. When there was a lack of social support, some people were unable to attend their appointment. Patients had shared how they had appreciated support to make sure they understood instructions during their treatment and felt that their dentist understood their circumstances and treatment needs. Some stakeholders described how they voluntarily guided people to a dentist because they were afraid that the patient would not proceed with their visit or treatment:

There are some people who come very often when there is guidance, and they just have that as a regular part. If the counselling is not there, they don’t come either. And oral care is then also part of the whole counselling process. (R4, OHC professional)

#### Theme 6: QI of OHC

This theme included initiatives to improve quality in the wider sense, including accessibility to OHC. Such initiatives for QI varied across categories of stakeholders. A large part of QI of OHC was driven by informal care initiatives. For example, homeless shelters explained that they offered health consultation for their target group and worked together with other stakeholders to have clients receive basic OHC. The emergency fund explained that they actively raised funds to provide financial support for OHC treatments. Médecins du Monde explained that local informal care, provided by volunteer dentists, was effective while the organization was actively involved at a location. However, once their interventions end, this informal care gradually diminished, highlighting Médecins du Monde’s role as a key facilitator. Therefore, they have launched a compassion network to make sure that informal care is organized on a structured basis across the country. Other stakeholders mentioned that they are involved in QI by sharing information about OHC with people:

I have sometimes told people, you know children can go to free oral care, things like that, I have said that to people. (R10, social and welfare worker)

When looking at formal care, the municipality described improving outreach and information to the target group, by providing information about treatments and collective municipality health insurances with OHC coverage. The municipality also worked to bring multiple parties together, as they were in a good position to keep contact with all stakeholders. OHC providers involved in special care explained that they were mostly involved in the improvement of care for anxious patients and the quality of the educational level of staff by providing training opportunities. Next to this, they worked on development of oral care instructions adjusted to the target group, such as instructions with pictures instead of written documents and paperwork instead of digital solutions:

I have lists with pictures of toothbrushes on them and then patients can just stick them on a door and then they can put a cross by the day if they have brushed their teeth, very old-fashioned but that works well. They like paper cards, so then we have nothing to do with a lot of digital stuff; at least in my experience, they are still of that old-fashioned mindset. (R4, OHC professional)

Stakeholders agreed that more work was needed to improve the quality of care through raising awareness about oral health problems within organizations. This would enable patients to be better informed about OHC. According to stakeholders, basic care should be available to everyone, and informal care networks of volunteer dentists needed to be kept alive until a more structured solution is established. Stakeholders agreed that people should not rely on OHC from emergency funds and medical volunteers:

We could, for instance, also take a look at: how do we get people to, how can we deploy people, the supporters, to come to that oral care a bit more. But then of course something has to be offered, within the basic package. . . . If people don’t have money and can’t pay for it, I can mobilize them, but I have nothing to offer, I’m just empty-handed and they’re just disappointed. (R10, social and welfare worker)

Stakeholders also expressed their concerns and risks for QI of care and accessibility of care. They were particularly concerned about the risk of emerging shortages of OHC providers and the potential increased need for care when structural solutions were established. Social workers described being often overburdened with work but that they realized manpower was essential for the success of new initiatives. Social workers found it difficult to get people to visit a care provider and that people sometimes distrusted initiatives that are linked to the system:

Health and healthy lifestyle interventions are typically driven by political initiatives. In our welfare organization, our assignments also come from the municipality. However, if these initiatives are perceived as coming “from the system,” people tend to resist them and refuse to engage. (R10, social and welfare worker)

#### Theme 7: Diversity of target group

Professional stakeholders described the diverse backgrounds and characteristics of the citizens they work with, such as debt clients, patients with dental anxiety, homeless people, those with low education or limited health literacy, people with financial constraints and migrant workers, or undocumented people. Stakeholders explained that these citizens often experience complex social problems, with multiple issues frequently overlapping. For example, some people are chronically ill, and most stakeholders mentioned that their target citizens face general health problems. According to stakeholders, people make unhealthy choices because these are more convenient and affordable compared with maintaining a healthy lifestyle or they have a cultural background with different traditions and beliefs compared with Western society. Because of multiple challenges, stakeholders stated that OHC is not always a priority for this group:

On several levels, you think much healthier choices can be made in this respect because at the Emergency Fund of course you get to see the receipts, what everyone buys. That you think: oh boy, if you really have no money, and must feed three children from that, is this really the best choice? For example, lots of sweets and biscuits, but also just the deep-fat, frozen and deep-fried things. You understand that, because that’s cheap, and children eat that easily too. (R7, social and welfare worker)

Stakeholders also described the varied social support available for the target groups, which included a substantial amount of help and support in low-income communities through social networks. These networks were important for oral health behavior and for seeking care. Stakeholders had also encountered people who had no social support:

You really have people who really do have a good network, that’s kind of the only group who either have good care or good outpatient support or a partner. But I also find it very distressing to see many patients who come for anaesthesia and then someone must go home with them. And that they then say: I don’t have anyone; I really do see that as loneliness. (R3, OHC professional)

#### Theme 8: Understanding of OHC

Stakeholders understood OHC as a broad definition of self-care, professional (preventive) care, and as care that should be available to anyone who needed it and not just to those who could afford it. Stakeholders described a difference between OHC and basic OHC. Basic OHC included pain relief measures, restoration of front teeth to enable smiling, restoration of chewing function, and resolution of inflammation, ultimately promoting better self-care:

I think overall that oral care should be accessible to everyone. At a basic level that people are pain- and inflammation-free; and that regardless of finances, it is just care that you are allowed to use as a Dutch person in a well-functioning healthcare system. (R3, OHC professional)

Basic care means no whole renovations and no expensive treatments like crowns, bridges and implants; so it’s really about basic oral care to be pain-free and inflammation-free and be able to eat again. You may well be missing a few molars then. But that you will be able to eat normally. (R9, social and welfare worker)

In contrast, stakeholders’ understanding of good quality OHC varied. Some felt that good quality OHC existed only if people were able and willing to pay for it. Others felt that good-quality OHC meant that an OHC provider provided care of objectively good quality. Good-quality OHC was also perceived as something that had positive effects on people’s lives and fulfilled an educational role. Stakeholders agreed that good-quality OHC would also mean that care was accessible and preventive:

Well then, on the one hand, I think qualitatively in terms of content, the treatments, I think, so that someone with a dental problem is helped with a treatment, or indeed with a cleaning, that the goal you have with that treatment is also achieved. But on the other hand, I also think, in terms of policy, that access to oral care affects the quality of oral care itself. (R7, social and welfare worker)

Stakeholders shared their perspectives about opportunities for QI of OHC: that prevention of oral diseases was not given enough attention yet within the target group of citizens. They felt that self-care and oral health behaviors depended on people’s upbringing and social learning and that improving patients’ self-care was difficult. Social and welfare workers have witnessed people with good self-care who had better oral health:

It’s just hard for me to say whether oral health is culture or environment or the position they’re in. Because their position is often quite difficult, but I do see big differences with people who really take good care of themselves or let it go completely or maybe don’t quite know or maybe don’t have complaints. (R6, social and welfare worker)

### Social Worlds/Arenas Map

**Figure 3.**
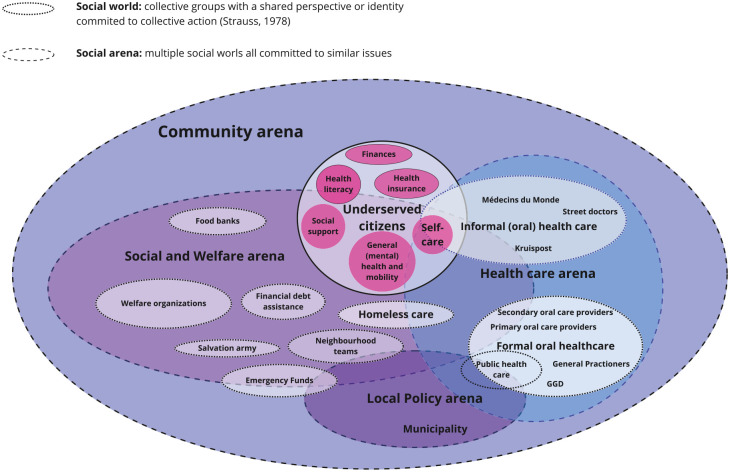
Social worlds/arenas map: major actors around underserved citizens on community level, based on the professional stakeholder perspectives.

A social worlds/arenas map (Fig. 3) was created drawing on the themes to visually organize the actors and entities involved in supporting the oral health needs of underserved citizens at the community level. The social world was structured into overlapping arenas: Community, Social and Welfare, Health Care, and Local Policy. At the center were underserved citizens, whose personal factors (e.g., finances and health literacy) influenced their oral health and navigation within the community. These factors were represented in circles around them. The Social and Welfare arena included support services such as welfare organizations, financial debt assistance, and emergency funds, which often collaborated with other arenas, including informal OHC providers from the Health Care arena. The Health Care arena comprised both formal and informal health care providers, highlighting contributions to improve access to OHC for the underserved. A successful implementation of community engagement was given by the homeless shelter who mentioned that the local dental school involvement resulted in more awareness of oral health within the homeless shelter. Public health care was also included in this arena, emphasizing the broad network needed to address health disparities. Finally, the Local Policy Arena is part of the Community Arena, as it involved local governmental and policy-making entities who were involved in shaping the supportive landscape for underserved citizens. Compared with other arenas, the Local Policy Arena does not have direct contact with underserved citizens when it comes to supporting the oral health need of the underserved at the community level. However, they did influence the OHC needs indirectly through their collaborations with other social worlds, such as emergency funds and public health care. The overlap between arenas illustrated interconnections among different sectors and emphasized how these entities worked together to provide comprehensive support in OHC. The Social and Welfare Arena showed the most interactions and emerged as a powerful arena in supporting underserved citizens at the community level. Furthermore, the overlaps of arenas and social worlds (Fig. 3) highlighted the importance of multistakeholder engagement in efforts for improving OHC for underserved citizens at the community level.

### Positional Map

After analyzing the interviews, positions regarding the importance of QI of OHC and stakeholders’ efforts in QI were formulated based on viewpoints and quotes from stakeholders who responded in the interviews:

Position 1: Municipal-specific OHC policy does not exist; it is part of existing health and poverty reduction frameworks.
Position 2: A supportive system should be formalized to facilitate access to OHC of good quality.
Position 3: There is unclarity about ownership of OHC accessibility.
Position 4: Collaboration between social and welfare organizations and OHC providers raises awareness of OHC.
Position 5: Emergency funds wish to collaborate more with OHC providers.
Position 6: Within social organizations, OHC is one of multiple problems and not the priority.
Position 7: Basic OHC should be available for everyone.
Position 8: Without support from a personal social network, OHC is difficult to access.
Position 9: The quality of OHC needs to improve for underserved groups.
Position 10: The shortage of OHC providers is a risk for quality of OHC.
Position 11: Collaborations between OHC providers and social and welfare organizations are needed to improve OHC quality for underserved people.
Position 12: Good-quality OHC means that OHC is accessible for all and is preventive.
Position 13: Good-quality OHC is a privilege and available only to those who can (financially) afford it.

**Figure 4.**
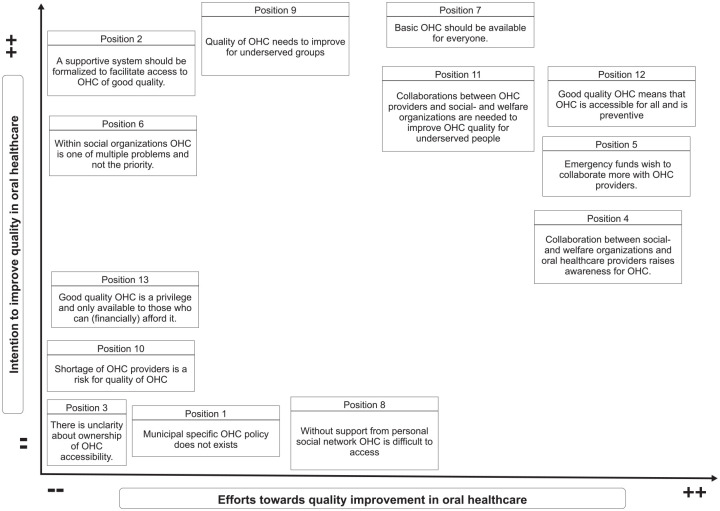
Positional map on positions derived from interviews with community-level professional stakeholders.

The positional map (Fig. 4) shows the positions taken by community-level stakeholders on topics and issues as discussed from the interviews. This visual map categorized varying stakeholder positions regarding OHC based on their perceived intentions and efforts. In Figure 4, the horizontal axis indicates the efforts toward QI in OHC. The vertical axis indicates the intentions to improve the quality of OHC. The overall picture shown is that most stakeholders express an intention to improve the quality (including accessibility) of OHC, whereas a minority feel they or others make an effort to actually improve the quality of OHC. The outliers show that low intention and low efforts toward QI in OHC are mostly explained by the absence of clarity of ownership for accessibility of OHC and that no specific municipal OHC policies were described by the stakeholders.

## Discussion

The field of stakeholders involved in OHC at the community level is diverse, and collaborations among stakeholders are common, with direct contact with citizens being a key aspect of their roles, particularly for social workers. Interestingly, this study revealed that stakeholders who were not directly involved in OHC provision did not specifically prioritize oral health.

These findings concur with previous research that described that MHS had a positive attitude about OHC but paid little attention to improving oral health, due to lack of time, lack of oral health knowledge, and other pressing issues from clients (van Spreuwel et al. 2021). Although no stakeholder group considered themselves as the main responsible actor, they all were involved in some QI efforts. Initiatives to increase OHC accessibility were mainly described by the informal OHC providers, such as Médecins du Monde. It is notable that QI initiatives at the community level evolve around the accessibility of OHC. Stakeholders were concerned about the accessibility of OHC for citizens with whom they work, mentioning financial position and health insurance as key determinants. The stakeholders’ first priority was that at least basic OHC should be financially accessible for everyone. Further priorities included a clear need for simplified access to OHC and strengthening social support. Those stakeholders who recognized reasons to prioritize accessibility of oral care often felt a lack of support within their organizations. Access to OHC was also found difficult without social support from professionals, volunteers, and citizens’ relatives and friends. While stakeholders agreed that people should not rely on emergency funds and volunteers, they found it unclear what organization or individual held the ownership and responsibility for access to OHC.

The situational analysis provided a starting point to inform health policy and shows that stakeholder values are important for problem solving and evidence-informed oral health policy making. This is important because efforts to improve QI need action from both OHC providers and social and welfare workers to support people who avoid care but who will still be in close contact with social organizations. To place the findings of the present situational analysis into context, these findings should be discussed alongside an existing framework for oral health systems improvement (Listl et al. 2023). Normative goals shared by stakeholders included consensus that OHC accessibility is limited and, therefore, access should rely on a supportive system structure rather than individual efforts or voluntary work providers. While emphasizing the need for basic care availability to everyone, stakeholders advocate for the preservation of informal care networks until a more structured solution is established. When looking at health systems arrangements considered relevant by stakeholders, financial arrangements were most discussed. Financial reasons seem to be a recognized barrier to accessibility of OHC in other high-income countries, such as the United States and Canada (Thompson et al. 2014; Vujicic et al. 2016). For Europe, the World Health Organization (WHO) has identified dental care as a key driver of catastrophic health spending (WHO Regional Office for Europe 2023). It must be noted that next to this, nonfinancial barriers can play an important role, such as dental anxiety, perceived stigma, and struggles to attend appointments (Carlsen et al. 2021). Further, social networks play a vital role in oral health behavior and seeking care, with variations observed in social support levels among individuals (Dahlan et al. 2019). One of our most striking and consistent findings was the lack of clarity around governance arrangements and ownership and responsibility for accessibility of OHC. Stakeholders do not seem to realize that the PHA was introduced in the Netherlands in 2008, providing a legal reason for municipalities to support the prevention of (oral) health diseases through MHS (Middlekoop et al. 2016). At the least, the implementation of this Act seems to encounter barriers. Stakeholders conceptualize delivery arrangements in terms of high-quality OHC being dispensed primarily within the confines of the dental clinic. Fewer stakeholders, especially those who do not have OHC as their primary focus, acknowledge the role and shared responsibility of community actors in creating and fostering opportunities for citizens to lead healthy lives, thereby reducing the risk of future oral diseases and maintaining oral health beyond the scope of clinical dental care. Stakeholders find implementation strategies targeted at consumers important, especially information and education on self-care and the importance of maintaining good oral health.

How do these findings support future work? The findings of this situational analysis provide relevant information for enhancing collaboration among community-level stakeholders to improve the accessibility and quality of OHC. In addition, this study can help to raise awareness among stakeholders about their roles in OHC at the community level and identify obstacles and solutions for change such as the perceived lack of importance within their organization, competing priorities, and discomfort discussing oral health with clients as a non-OHC provider. Likely solutions include actions to address allocation, not just of resources but of responsibilities—in other words, governance. In particular, the findings suggest that enhanced communication and collaboration between OHC providers and other community-level stakeholders could be instrumental in addressing these barriers. For example, integrating OHC considerations into existing community networks focused on improving the general health of vulnerable populations could enhance interprofessional communication. This would require inviting OHC providers to community networks, ensuring that oral health is addressed alongside health and social concerns. I addition, dental training programs at academic institutions could encourage dental students to contribute to community networks through community service activities, fostering early collaboration with community stakeholders. Another strategy to enhance access to OHC in Amsterdam is to adopt the Sociale Tandarts (Social Dentist) initiative from Rotterdam, the Netherlands (Sociale Tandarts Rotterdam 2024). The program is designed to improve access to OHC for vulnerable and underserved populations by providing free or subsidized dental services. This initiative is driven by volunteer dental professionals who donate their time and expertise to serve the community. By implementing a similar model, Amsterdam could address gaps in OHC access, ensuring that disadvantaged groups receive dental treatments and preventive care.

### Strengths and Limitations

Strengths of this study include its thoroughness in identifying and recruiting stakeholders involved in OHC at the community level that go beyond health care providers. By including social workers, public health experts, and municipal policy makers among health care providers, diverse professional perspectives and their interactions at the community level became visible. The use of grounded theory allowed for the emergence of themes from data, enhancing the study’s credibility and reliability. The adoption of the situational analysis method provided a framework for investigating and mapping the complex circumstances surrounding QI efforts in OHC at the community level. This approach enables a comprehensive understanding of all actors as well as the contextual factors influencing oral health initiatives in Amsterdam, the Netherlands.

The limitations of this situational analysis were the use of a convenience sampling method, which may have resulted in interview respondents among non-OHC stakeholders who paid more attention to oral health compared with their counterparts. Readers should exercise caution regarding the transferability of the qualitative findings beyond the region of Amsterdam, the Netherlands, as the sociocultural and health care system dynamics in Amsterdam may not be representative of other communities within the Netherlands or other countries. However, limited funding for OHC among underserved people is a wider global problem (WHO 2022), which makes the results still useful to other regions and countries. Efforts were taken to address researcher reflexivity to ensure trustworthiness, rigor, and dependability, as the researcher’s (S.B.) background in dentistry may have shaped the interview questions or data interpretation. To mitigate this risk, the team used a reflexive approach—documenting reflections after each interview and discussing coding decisions with other researchers. It must also be noted that the interviews were conducted in Dutch and later translated into English for data analysis. While there is always a risk of losing certain cultural or linguistic nuances, we have taken steps to minimize such loss, including manually reviewing translations.

The findings of this situational analysis will support the next phase of the project in the design process of a deliberative co-creating phase focusing on priorities and needs for improvement of OHC quality at the community level by the research team within the DELIVER project. Before further collaborative steps with professional stakeholders can be taken, the perspective of citizens associated with the interviewed organizations should be researched. Patient perspectives were not captured in this initial phase, which centered primarily on community-level and professional viewpoints. The plan in subsequent research is to incorporate patient voices through a participatory approach, ensuring a more comprehensive understanding of OHC experiences and needs.

## Conclusion

This situational analysis provided an understanding of priorities and interactions of key stakeholders involved in QI initiatives at the community level for underserved adults in Amsterdam, the Netherlands. It highlighted the complex landscape of stakeholders, their diverse priorities, and understanding of the role of OHC including challenges in effectively addressing oral health accessibility. The lack of clarity regarding ownership and responsibility necessitates a more coordinated allocation of roles and responsibilities. Stakeholders, including those not directly involved in OHC provision, could play important roles within all aspects of quality of OHC. The call for the availability of basic care for everyone reflects a shared vision for a more inclusive and structured OHC system. At the same time, this situational analysis shows that meeting financial needs cannot be the sole solution, if social support and environmental factors were to be neglected.

## Author Contributions

S. Begovic, contributed to conception, design, data acquisition, analysis, and interpretation, drafted and critically revised the manuscript; M.W. van der Linden, contributed to conception, design, data acquisition and interpretation, critically revised the manuscript; K. Rosing, M. Lorenz, S. Listl, contributed to conception, design, data interpretation, critically revised the manuscript; L.E. de Almeida, contributed to design, data interpretation, critically revised the manuscript; M.H. van der Veen, contributed to conception and design, data acquisition, analysis, and interpretation, critically revised the manuscript. All authors gave final approval and agree to be accountable for all aspects of the work.

## Supplemental Material

sj-docx-1-jct-10.1177_23800844251332227 – Supplemental material for Whose Responsibility Is It? A Community-Level Situational Analysis of Oral Health Care in Amsterdam Supplemental material, sj-docx-1-jct-10.1177_23800844251332227 for Whose Responsibility Is It? A Community-Level Situational Analysis of Oral Health Care in Amsterdam by S. Begovic, M.W. van der Linden, K. Rosing, L.E. de Almeida, M. Lorenz, S. Listl and M.H. van der Veen in JDR Clinical & Translational Research
