# Supplementary material for: Whose Responsibility Is It? A Community-Level Situational Analysis of Oral Health Care in Amsterdam
Source: JDR Clin Trans Res. 2025 May 2;11(2):181–94. doi: 10.1177/23800844251332227 (PMC12967396; doi:10.1177/23800844251332227)
Supplement: sj-docx-1-jct-10.1177_23800844251332227 – Supplemental material for Whose Responsibility Is It? A Community-Level Situational Analysis of Oral Health Care in Amsterdam [file sj-docx-1-jct-10.1177_23800844251332227.docx]

**APPENDIX FILE**

**Whose responsibility is it? A community-level situational analysis of oral healthcare in Amsterdam.**

Sehida Begovic, Michiel van der Linden, Kasper Rosing, Linnea Eisemann de Almeida, Michael Lorenz, Stefan Listl, Monique van der Veen

**Appendix: Table 1**

**Table 1. Respondents by stakeholder category and organization**

| **Respondent number ®** | **Stakeholder category** | **Organization** |
| --- | --- | --- |
| R1 | Public health care professionals | Municipal Health Services Amsterdam (GGD) |
| R2 | Municipality policy makers | Municipality of Amsterdam, Collective Health Insurance for Minimum Income Groups and Poverty Reduction (Gemeente Amsterdam, Collectieve Zorgverzekering voor Minima en Armoedebestrijding) |
| R3 | Oral health care professionals | Anxiety dentist from SBT Special Dentistry (Stichting voor Bijzondere Tandheelkunde) |
| R4 | Oral health care professionals | Dentist with affinity for Special Dentistry |
| R5 | Primary and secondary health care professionals | Social nurse at Streetdoctors |
| R6 | Social and welfare workers | Homeless shelter (de Regenbooggroep) |
| R7 | Social and welfare workers | Emergency Fund (Fonds Bijzondere Noden Amsterdam) |
| R8 | Social and welfare workers | Buurtteam branch (social assistance for care, housing, health, income, financial issues, meeting people, and safety). |
| R9 | Social and welfare workers | Médicins du Monde (Dokters van de Wereld) |
| R10 | Social and welfare workers | DOCK Welfare institution |

**Appendix: Figure 1**

**Figure 1. Ordered situational map of actors involved in quality improvement efforts within oral healthcare on community level in Amsterdam, the Netherlands.**


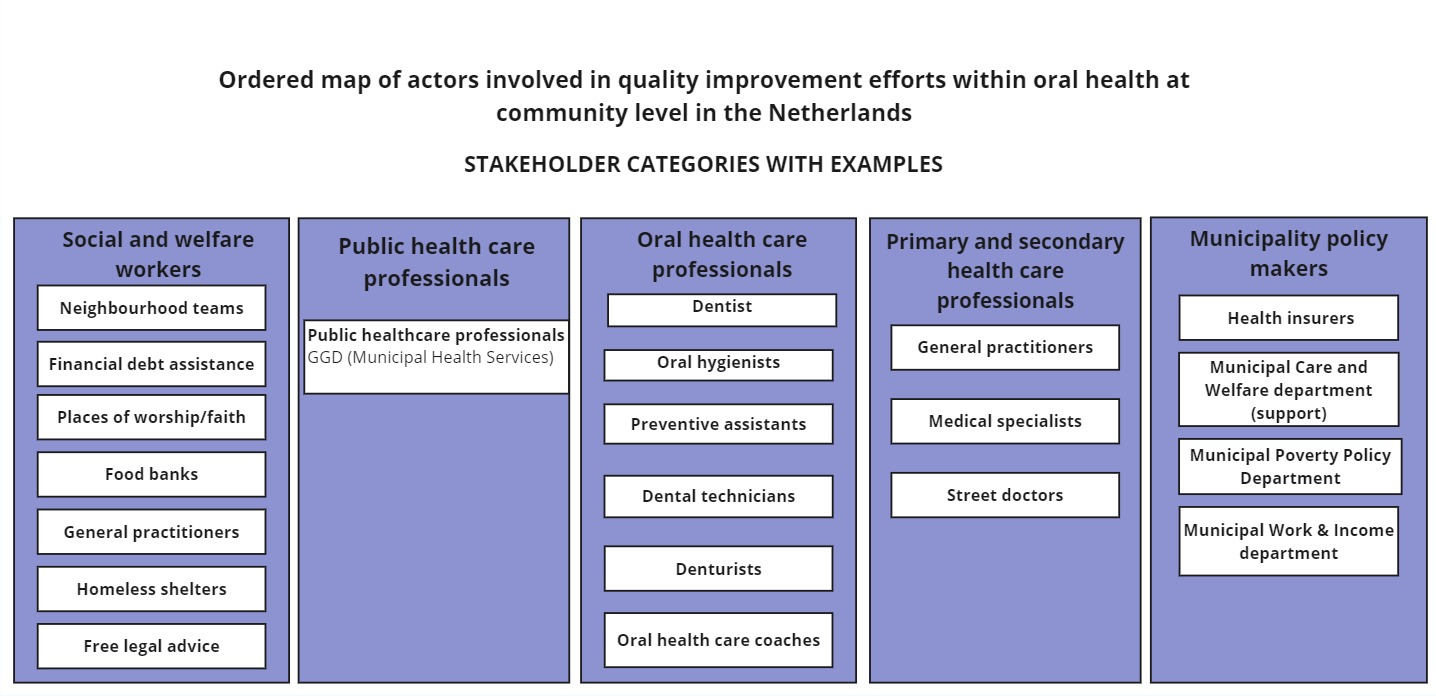


**Appendix: Figure 2**

**Figure 2. Situational map: relations between actors involved in quality improvement of oral healthcare at the community level in the Netherlands.**


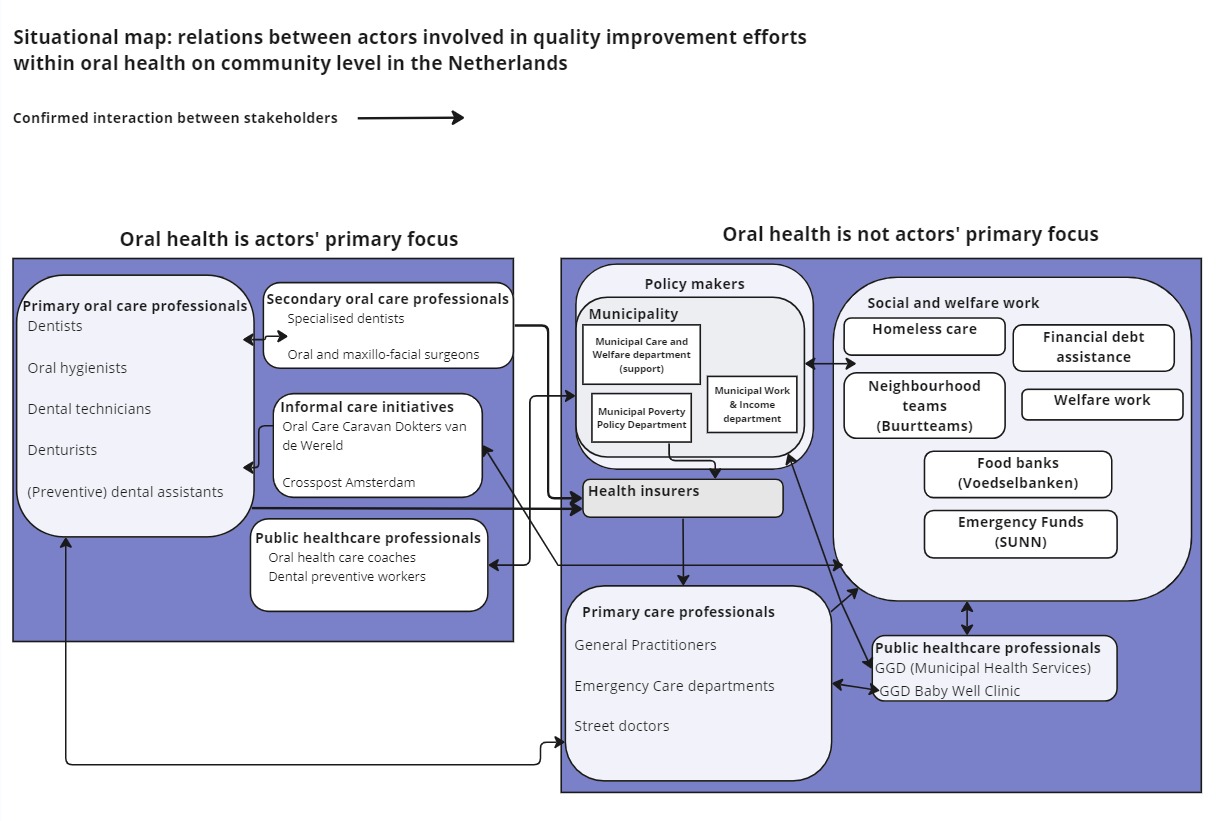


**Appendix: Figure 3**

**Figure 3. Social worlds/arenas map: major actors around underserved citizens on community level, based on the professional stakeholder perspectives.**

**
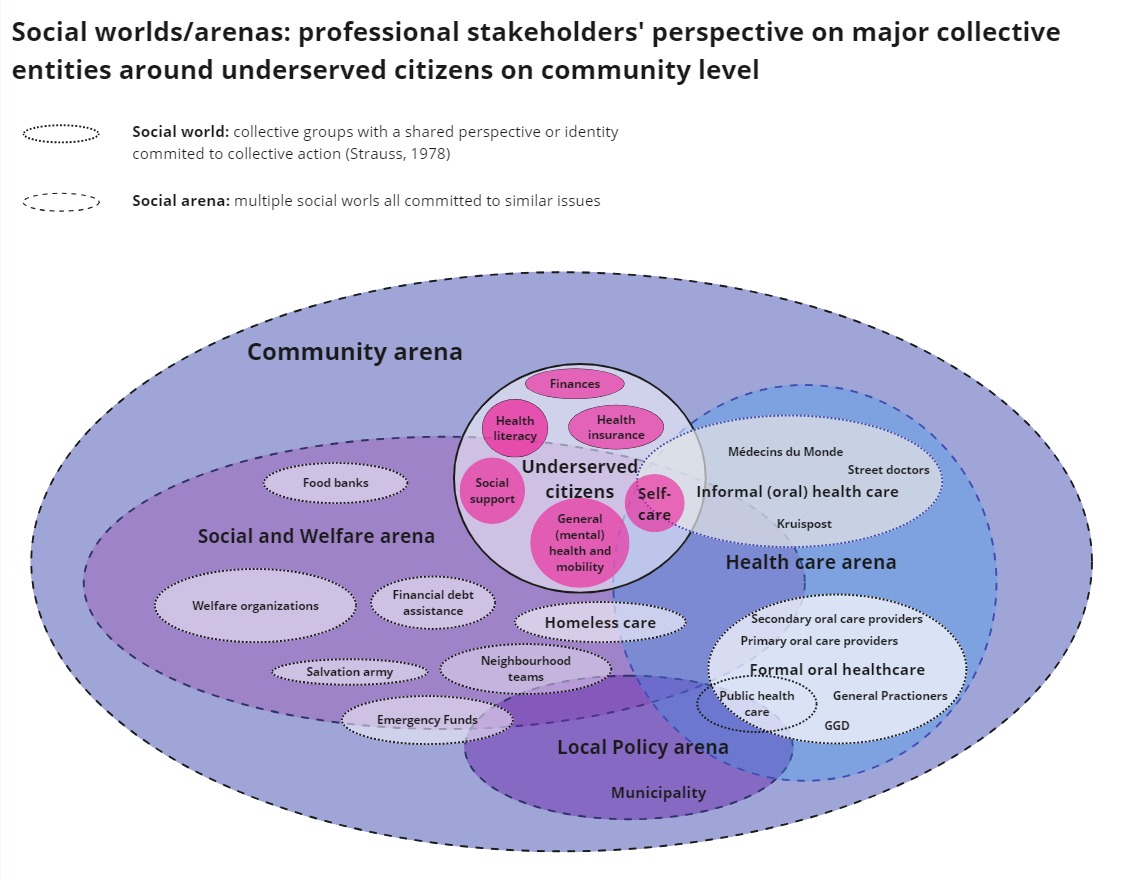
**

**Appendix: Figure 4**

**Figure 4. Positional map on positions derived from interviews with community-level professional stakeholders**


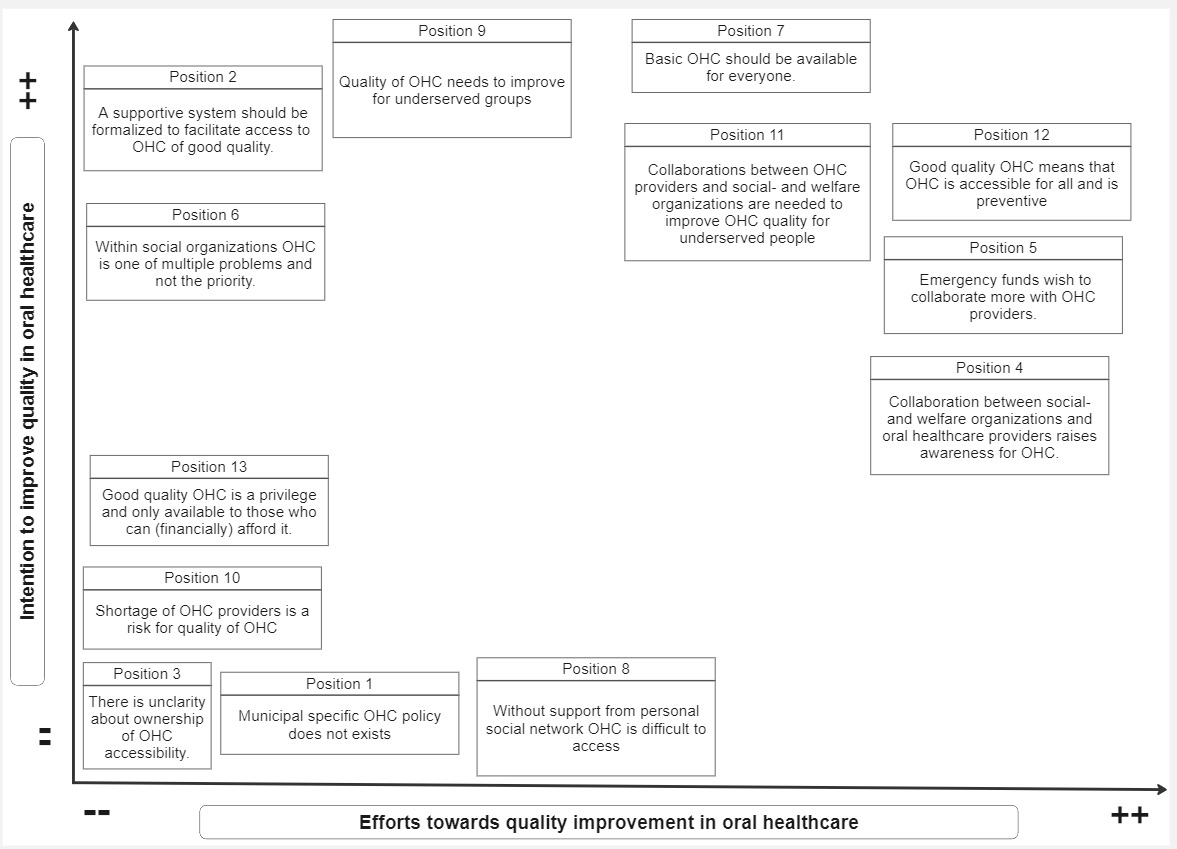


| **Topic** | **Main question** | **Specific questions** |
| --- | --- | --- |
| **Introduction - General topic: *What is good quality oral health care?*** | How can accessibility and quality of oral healthcare be improved in vulnerable people/situations on community level | Who and what matters, and what (elements) make a difference? |
| **Initial general questions** | What job do you have within your organization?  What kind of people do you assist in your work and how? | For how long have you been working in this job? What attracted you to doing this work? What questions bring people to you? |
| **Understanding/definition oral health care** | What is your understanding of oral healthcare /oral health care? What should oral health care look like? | What would quality mean in this sense?  Can you give an example? |
| **Priority of Oral healthcare** | Is oral health discussed with your clients and how? Do you ask about oral health yourself? Is oral health given attention to? Is prevention, selfcare, or lifestyle part of conversations? If yes, which forms of selfcare? Does this include oral healthcare and experiences, needs, perspectives regarding oral health? Is health literacy discussed? Do you feel equipped/ do you have enough opportunity to discuss oral health? | If health issues are discussed, what priority do they have for people? And what priority do they have for the social/public health workers?  Can you provide examples of these experiences, needs and perspectives?  What level of health literacy is seen?  What would you need/ what would help you to make you feel more equipped? |
| **Efforts** | What does your organization do to improve oral health care for your client-group? | What would you like your organization to do? What keeps your organization from doing more or differently? |
| **Patients’ needs and resources**  **(including Avoidance of oral healthcare)** | Do people mention or ask for help regarding (oral) health issues? What makes people refrain from discussing these issues? Is use and access to oral healthcare, or avoidance of oral healthcare discussed? | What (oral) health issues are part of intake or conversations? What problems/barriers in use or access or oral health care do you see? What is needed? What are potential solutions to avoidance of oral healthcare that are seen? Which barriers are seen? |
| **Opportunities for change** | What is the current situation? Do you see opportunities for changes? Do you feel there is a need for change? How could equitability and quality of care change? | Which barriers and opportunities do you See? In what direction should the situation change? What should or could be changed to improve oral health care? |
| **External policies & Incentives** | What kind of local, regional, or national factors have an influence on efforts to improve access to dental healthcare? | Can you mention specific performance measures, policies, regulations, or guidelines? How do they affect your efforts? |
| **Learning climate** | Can you describe a recent improvement initiative or an implementation of a new program regarding dental health care? | To what extent do you/your organization feel you can try new things to improve dental healthcare for vulnerable populations? Can you elucidate with and example? |
| **Culture** | To what extent are new ideas embraced and used to make improvements in your organization? | Can you give an Example? Would you rather characterize your organization as ‘open’ or ‘closed’ (e.g., toward people in-/outside the organization)? |
| **Field/community** | What is happening in the field of community-level oral healthcare? What cooperation exists? What are the most relevant organisations / actors regarding inclusive oral healthcare? | Are you aware of specific initiatives, resources or services? How do you work with them? How do specific policies/guidelines play a role in the situation regarding oral healthcare on community level? |
| **Own organisation/network, including**  **priority and flexibility regarding new oral health community initiatives** | How do people you work with feel about oral health? What help/hindrance do you experience in the context of your organization while putting oral health on the agenda? | To what extent is time/space available in your organization for oral healthcare initiatives? What policies work in favour / against oral health care delivery? |

**Appendix: Interview guide**

**Appendix: Interview reflections forms**

**Reflection form DELIVER Situational Analysis on Community level**

*Please complete this reflection form after each semi-structured interview before analyzing the interview.*

**Date of Interview:**

**Interviewer:**

**Interviewee:**

**Interview Preparation:**

Did you adequately prepare for the interview by researching the topic and the interviewee?

……………………………………………………………………………………………………………………………………………………………

Did you adjust the set of general open-ended questions to guide the interview?

……………………………………………………………………………………………………………………………………………………………

**Interview Process:**

How were you able to create a comfortable and relaxed environment for the interview?

………………………………………………………………………………………………………………………………………………………

Did you follow the semi-structured interview format while allowing flexibility for additional questions?

……………………………………………………………………………………………………………………………………………………….

**Questioning Techniques:**

How did you use active listening skills to understand the interviewee's responses?

……………………………………………………………………………………………………………………………………………………….

How did you probe further when necessary to explore deeper into certain topics?

……………………………………………………………………………………………………………………………………………………….

**Engagement of the stakeholder:**

How was the interview received by the interviewee and did the interviewee feel comfortable and engaged during the interview?

……………………………………………………………………………………………………………………………………………………..

Did they provide detailed and informative answers to your questions? If not, what could have been the reason?

………………………………………………………………………………………………………………………………………………………..

Were there any moments where the interviewee seemed hesitant or unresponsive?

………………………………………………………………………………………………………………………………………………………..

What questions did the stakeholder have before, during or after the interview?

…………………………………………………………………………………………………………………………………………………………

**Adaptability:**

How were you able to adapt the interview approach based on the interviewee's communication style and preferences?

……………………………………………………………………………………………………………………………………………………………

Did you make adjustments to the order or phrasing of questions when needed?

…………………………………………………………………………………………………………………………………………………………….

**Time Management:**

Were you able to manage the time effectively during the interview? If not, how can you improve this?

……………………………………………………………………………………………………………………………………………………………

Did you allocate sufficient time to cover all the planned questions/topics?

……………………………………………………………………………………………………………………………………………………………

**Self-reflection on your interview skills:**

What went well during the interview? What aspects of your performance were strong?

…………………………………………………………………………………………………………………………………………………………….

What areas could be improved upon for future interviews and how do you plan to do this?

…………………………………………………………………………………………………………………………………………………………….

What did you learn from this experience that could be applied to future interviews?

…………………………………………………………………………………………………………………………………………………………….

**Additional Comments/Notes:**
